# Space–time clusters for early detection of grizzly bear predation

**DOI:** 10.1002/ece3.3489

**Published:** 2017-11-29

**Authors:** Joseph Kermish‐Wells, Alessandro Massolo, Gordon B. Stenhouse, Terrence A. Larsen, Marco Musiani

**Affiliations:** ^1^ Environmental Design University of Calgary Calgary AB Canada; ^2^ Ethology Unit Department of Biology University of Pisa Pisa Italy; ^3^ Department of Ecosystem and Public Health Faculty of Veterinary Medicine University of Calgary Calgary AB Canada; ^4^ UMR CNRS 6249 Chrono‐Environnement Université Bourgogne Franche‐Comté Besancon France; ^5^ fRI Research Grizzly Bear Program Hinton AB Canada

**Keywords:** GPS, grizzly bear, SaTScan, Space–time clustering method, *Ursus arctos*, west‐central Alberta

## Abstract

Accurate detection and classification of predation events is important to determine predation and consumption rates by predators. However, obtaining this information for large predators is constrained by the speed at which carcasses disappear and the cost of field data collection. To accurately detect predation events, researchers have used GPS collar technology combined with targeted site visits. However, kill sites are often investigated well after the predation event due to limited data retrieval options on GPS collars (VHF or UHF downloading) and to ensure crew safety when working with large predators. This can lead to missing information from small‐prey (including young ungulates) kill sites due to scavenging and general site deterioration (e.g., vegetation growth). We used a space–time permutation scan statistic (STPSS) clustering method (SaTScan) to detect predation events of grizzly bears (*Ursus arctos*) fitted with satellite transmitting GPS collars. We used generalized linear mixed models to verify predation events and the size of carcasses using spatiotemporal characteristics as predictors. STPSS uses a probability model to compare expected cluster size (space and time) with the observed size. We applied this method retrospectively to data from 2006 to 2007 to compare our method to random GPS site selection. In 2013–2014, we applied our detection method to visit sites one week after their occupation. Both datasets were collected in the same study area. Our approach detected 23 of 27 predation sites verified by visiting 464 random grizzly bear locations in 2006–2007, 187 of which were within space–time clusters and 277 outside. Predation site detection increased by 2.75 times (54 predation events of 335 visited clusters) using 2013–2014 data. Our GLMMs showed that cluster size and duration predicted predation events and carcass size with high sensitivity (0.72 and 0.94, respectively). Coupling GPS satellite technology with clusters using a program based on space–time probability models allows for prompt visits to predation sites. This enables accurate identification of the carcass size and increases fieldwork efficiency in predation studies.

## 1 INTRODUCTION

Predation and scavenging influence prey and predator population dynamics and community structure (Holt, 1977), but key elements of predation, such as frequency of predation, prey size, species, and sex, have proven difficult to quantify. Therefore, predator foraging ecology is often based on scat analysis that cannot always provide reliable data on these parameters (Fortin et al., 2013; Ripple & Larsen, 2000). For instance, prey sex and age usually cannot be determined from remains in scat, whereas visits to predation sites provide high‐quality estimates of food consumption as well as prey carcass characteristics (Cristescu, 2013), but as information available at a predation site deteriorates with time, prompt detection of the predation event is important to collect such key data as prey sex, age, carcass consumption (Cristescu, Stenhouse, & Boyce, 2014a; Rauset, Kindberg, & Swenson, 2012), and predator behavior.

**Figure 1:**
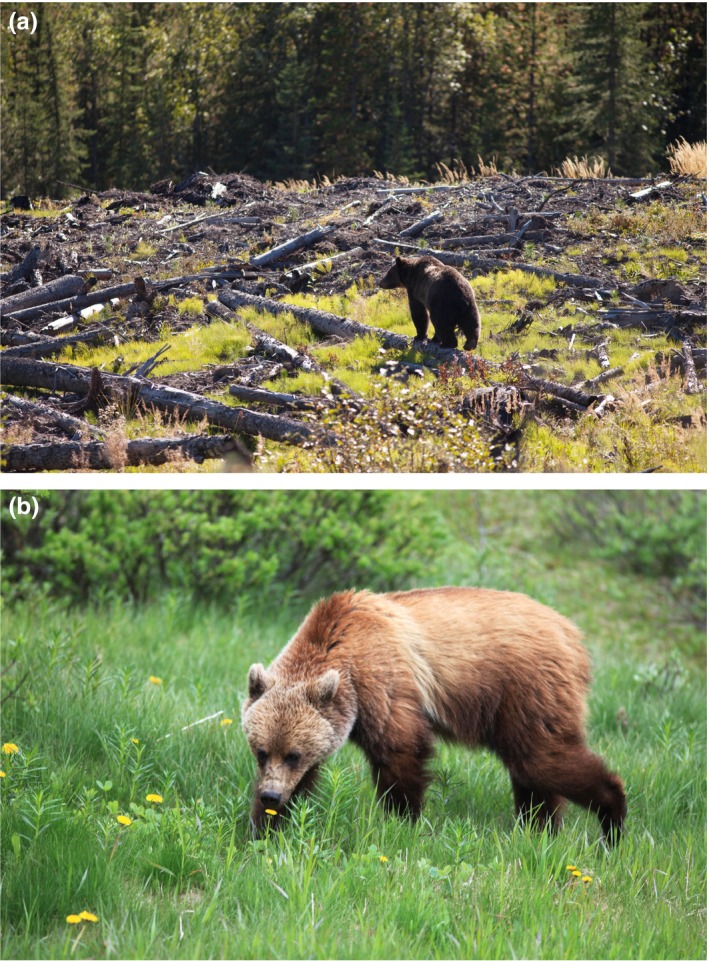
Study organism. (a) Grizzly bear *Ursus arctos* in a logging cut in the Kakwa region, Alberta, Canada; (b) Grizzly bear grazing

Animal tracking collars have been used for decades, and biologists have paired location data with field site investigations to explore associated natural history, including predation events (see for example, Jedrzejewski et al., 2000). However, the innovation of satellite transmitters has improved remote wildlife monitoring (Anderson & Lindzey, 2003; Franke, Caelli, Kuzyk, & Hudson, 2006; Knopff, Knopff, Warren, & Boyce, 2009; Sand, Zimmermann, Wabakken, Andrèn, & Pedersen, 2005; Tambling, Cameron, Du Toit, & Getz, 2010; Webb, Hebblewhite, & Merrill, 2008) and has improved our ability to detect predation sites. Global Positioning System (GPS) collars allow for animal movements to be monitored at a predefined frequency, providing a series of locations at regular intervals (Anderson & Lindzey, 2003; Franke et al., 2006; Sand et al., 2005; Zimmermann, Wabakken, Sand, Pedersen, & Liberg, 2007). The purpose of our research was to improve the detection of predation events for grizzly bears (Figure 1) using animal tracking collar data to reduce the delay between predation events and site visits.

Technological developments in wildlife radio‐tracking have improved our ability to detect predator kill sites. Kill sites were primarily detected by visually locating blood and tracks in the snow during ground and helicopter surveys (Knopff et al., 2009; Kunkel, Ruth, Pletscher, & Hornocker, 1999) as well as by aerial telemetry tracking of collared animals. In some cases, predation sites were investigated after researchers identified clusters of locations, but the efficiency of data collection was partially compromised by delayed site visits because location data were obtained after collar retrieval or infrequently from ultra‐high‐frequency (UHF) collar remote downloads (Anderson & Lindzey, 2003; Webb et al., 2008). Cristescu, Stenhouse, and Boyce (2014b) studied GPS‐UHF collared grizzly bears, but only visited sites that were occupied by the predator at least a month before the data were downloaded. Such studies that relied on site investigations occurring well after the kill event often found scattered prey remains. While providing information on relative prey intake, this approach made species identification uncertain and missed predation of relatively small prey, and provided limited information on feeding behavior (Anderson & Lindzey, 2003; Franke et al., 2006; Sand et al., 2005; Webb et al., 2008). Satellite‐based GPS now permits kill sites to be detected in real time (Dahle et al., 2013; Rauset et al., 2012), resolving these issues and potentially enabling the study of multipredator systems that distinguish predation from scavenging (Knopff et al., 2009).

A growing practice is to visit GPS point clusters that are identified visually or using automated detection methods. Tools such as Python scripts defined clusters by constraining points in space and time (Cristescu et al., 2014b; Miller et al., 2013), k‐means clustering (VanMoorter, Visscher, Jerde, Frair, & Merrill, 2010), spatially joining buffered GPS points (Zimmermann et al., 2007), or spatial only noise (DBScan) clustering (Ebinger et al., 2016) have proven effective. When predators kill large prey or scavenge large carcasses, they often spend more time near to the carcass (Cristescu et al., 2014a; Knopff et al., 2009). This may create detectable space–time patterns in GPS data, characterized by relatively long periods spent by the predator in relatively small areas. The advent of GPS collars has made it possible to identify “clusters” with automated algorithms for grizzly bears (Cristescu et al., 2014b; Ebinger et al., 2016; Rauset et al., 2012) and other predators (Anderson & Lindzey, 2003; Knopff et al., 2009; Sand et al., 2005; Tambling et al., 2010, 2012; Webb et al., 2008). However, these automated approaches are not conceptually different from intersecting successive relocations, and there is an increasing need to quantitatively compare different approaches for detecting predation events and other features, for example, size of prey.

The aim of our research was to improve the detection of predation events using frequently downloaded grizzly bear location data to detect GPS location clusters, so allowing field visits more promptly than in past studies. Specifically, we aimed to build a protocol for early detection of predation sites for grizzly bears and to develop models to predict the presence of grizzly bear predation events and the size of prey carcasses found using solely spatiotemporal characteristics of GPS point clusters as predictors.

## 2 MATERIALS AND METHODS

### 2.1 Study area

**Figure 2:**
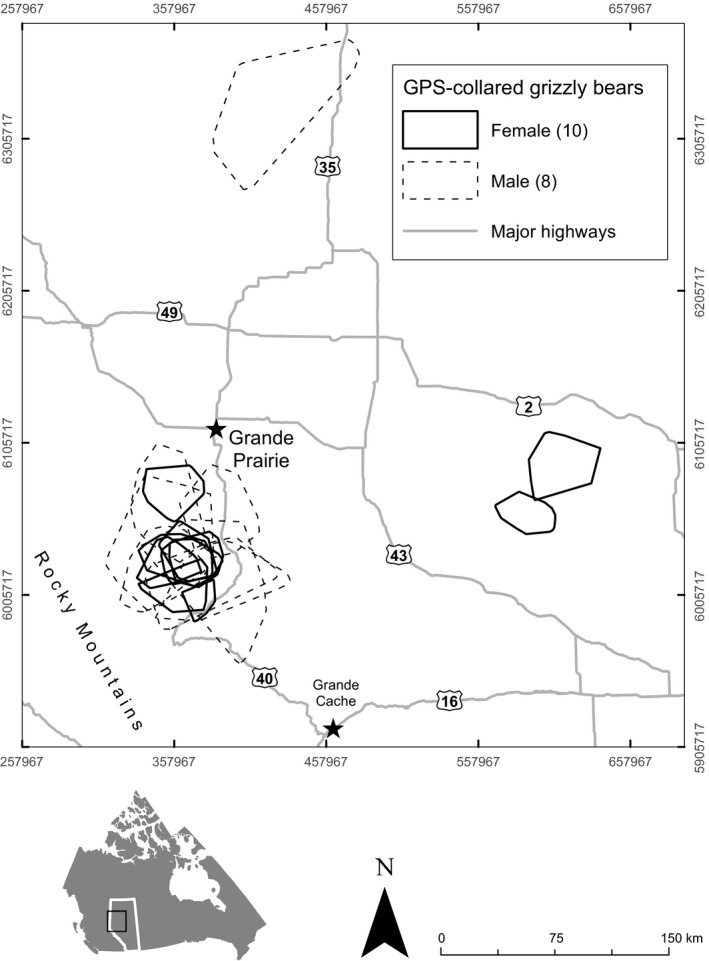
Study area for the 2006–2007 and 2013–2014 seasons indicated by individual ranges of male (dotted line) and female (solid line) GPS‐collared grizzly bears (*n *=* *18) tracked in west‐central, Alberta, Canada

Our study was largely conducted in 74 000 km^2^ area in the boreal forest along the eastern slopes of the Rocky Mountains, 85 km south of Grand Prairie in west‐central Alberta, Canada. Sites outside this area to the north and east were also occasionally used (Figure 2). Elevation ranged from 270 to 3280 m a.s.l. At lower elevations, the region was composed of montane, conifer, subalpine forests, and alpine meadows (Downing & Pettapiece, 2006). Wetlands and bogs were more common at lower elevations and to the north and east of the study area (Franklin, 2001).

Primary plant foods for grizzly bears in west‐central Alberta are sweet vetch (*Hedysarum alpinum, H. boreale* and *H. sulphurescens*) roots, cow parsnip (*Heracleum lanatum*), and clover (*Trifolium* spp.). Berries can be seasonally abundant and grizzly bears may forage on velvet‐leafed blueberry (*Vaccinium myrtilloides*), dwarf blueberry (*Vaccinium caespitosum*), buffaloberry (*Shepherdia canadensis*), and mountain huckleberry (*Vaccinium membranaceum*). Less abundant, but still present in grizzly bear diets, are lingonberry (*Vaccinium vitis*‐*ideae*), bearberry (*Arctostaphylos uva*‐*ursi*), and raspberry (*Rubus idaeus*) (Munro, Nielsen, Price, Stenhouse, & Boyce, 2006; Nielsen, McDermid, Stenhouse, & Boyce, 2010).

The dominant animal protein source in grizzly bears’ diets were ungulates as moose (*Alces alces*), white‐tailed deer (*Odocoileus virginianus*), mule deer (*Odocoileus hemionus*), elk (*Cervus canadensis*), and woodland caribou (*Rangifer tarandus*). Grizzly bears are not the only, nor primary predator in Alberta. They share the landscape with wolves (*Canis lupus*), cougars (*Puma concolor*), and black bears (*Ursus americanus*), all of which are known predators of ungulates (Ballard, Spraker, & Taylor, 1981; Ballard et al., 1979; Gustine, Parker, Lay, Gillingham, & Heard, 2006; Rauset et al., 2012; Swenson et al., 2007).

Human activity is dominantly resource extraction (forestry, oil and gas, open pit coal mining) with related linear habitat alterations including roads, pipelines, and seismic lines (White, Wulder, Gómez, & Stenhouse, 2011). The area is also used for recreational activities such as hunting, fishing, camping, hiking, and off‐highway vehicle use (Nielsen, Munro, Bainbridge, Stenhouse, & Boyce, 2004).

### 2.2 Grizzly bear location data

We used GPS collar data from 18 different grizzly bears captured and monitored during two different periods: 2006–2007 and 2013–2014 (Table S1). Bears were captured using aerial darting, leg‐hold snaring (only until 2009), and culvert trapping techniques (Cattet, Boulanger, Stenhouse, Powell, & Reynolds‐Hogland, 2008; Cattet, Christison, Caulkett, & Stenhouse, 2003).

Bears were equipped with Televilt/Followit GPS satellite collars (Followit, Televilt, Lindesberg, Sweden). Collars in both sampling periods were programmed to acquire hourly locations except for two collars deployed in 2006–2007. These were programmed with a 20‐minute interval and were aggregated to match the hourly data of the other collars. The primary difference in 2013–2014 compared to 2006–2007 was that animal location data could be downloaded via a satellite uplink rather than downloaded by aerial or ground‐based methods. Collars transmitted data via satellite every 10 hr during the nondenning period (April–December).

### 2.3 Study design

**Figure 3:**
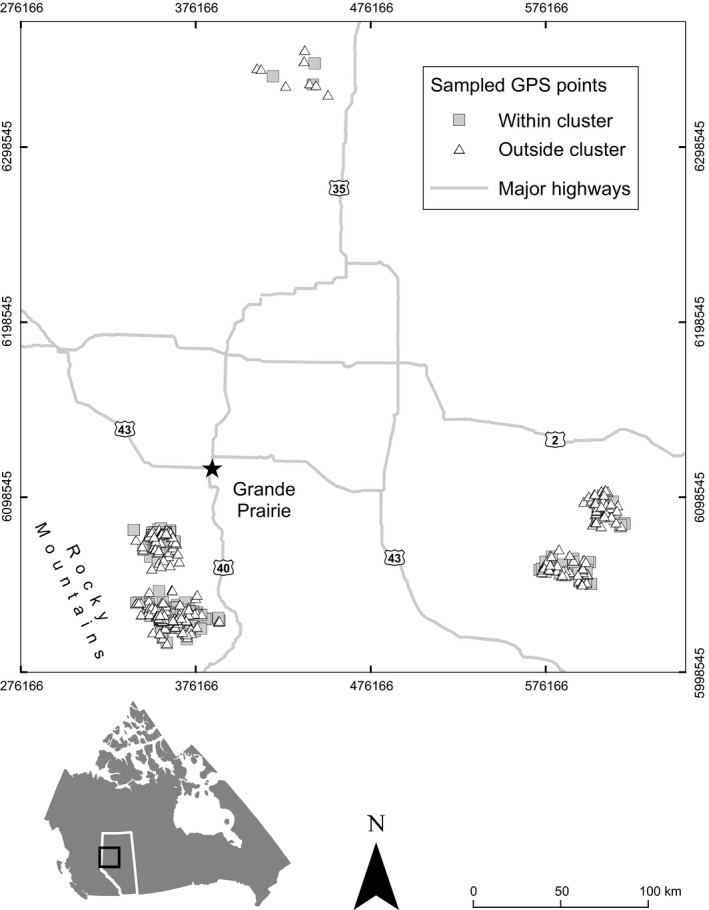
Sampled random GPS locations visited in 2006–2007 and labeled according to whether they fell within (gray squares) or outside of (white triangles) clusters. GPS clusters were generated *a posteriori*

**Figure 4:**
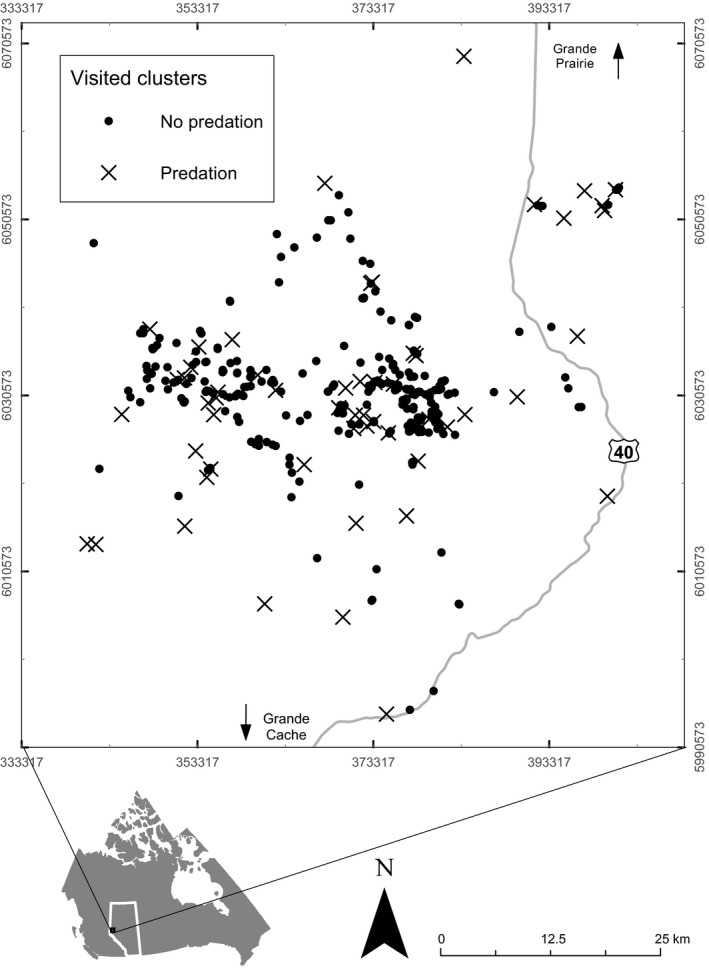
Visited STPSS clusters of GPS locations in 2013–2014 of collared grizzly bears tracked in the Kakwa region of west‐central Alberta, Canada. Sampled GPS cluster locations are shown and labeled according to whether a cluster was found and confirmed as predation event (X) and no predation (dot)

Our study was divided into three phases. First, we developed and tested a clustering method for detecting grizzly bear predation sites comparing random GPS points to clusters (Figure 3). Second, we developed and applied a field sampling design using the cluster detection method to identify high‐probability activity sites before field visit (Figure 4). Finally, we formulated models based on space–time cluster characteristics to detect predation or scavenging sites and prey carcass size.

### 2.4 Cluster detection protocol

To detect spatiotemporal clusters in the datasets collected at two different times, we used the retrospective space–time permutation scan statistic (STPSS) (Kulldorff, Heffernan, Hartman, Assunção, & Mostashari, 2005) in SaTScan 9.4 (Kulldorff, 2015). The STPSS selects event clusters (e.g., bear GPS points) by centering space–time cylinders on each event. The cylinder's base represents two‐dimensional geographic space, and its height represents time, in hours. The outcome is numerous overlapping cylinders considered to be potential clusters. The STPSS then applies a Poisson‐distributed probability function to compare the expected number of GPS points to fall in each potential cluster to the observed number of GPS points. This method allowed us to detect clusters of “cases” with distributions in space and time that potentially differ from an expected spatiotemporal pattern. For greater detail on the original development of SaTScan and STPSS, we direct readers to Kulldorff et al. (2005) and Kulldorff (1997).

We modified the STPSS's default search radius and temporal size used by Kulldorff et al. (2005) to be biologically relevant for grizzly bears (Table S2). This we achieved by setting the parameters according to studies on grizzly bear feeding behavior, on predation event characterization (Ballard et al., 1981; Rauset et al., 2012), and wolf predation (Webb et al., 2008). Values for maximum temporal size and maximum radius were defined according to a kill site handling time maximum of 7 days (rounded from 6 days to accommodate observed predation events of 1 kill/6.1 days) (Ballard et al., 1981) and a maximum radius of 50 m (Rauset et al., 2012). We considered only space–time clusters within the 95% confidence interval determined by SaTScan's Monte Carlo testing that compares the Poisson generalized likelihood ratio (GLR) for each cluster to the GLR obtained from 999 randomly simulated clusters (Kulldorff et al., 2005; Webb et al., 2008).

We ran the STPSS on 14‐day periods of individual grizzly bear GPS points. We chose two weeks based on a doubling of the seven‐day maximum grizzly bear occupancy of kill sites estimated by Ballard et al. (1981). Each dataset intentionally overlapped the consecutive period by 7 days to prevent exclusion or alteration of clusters that might potentially overlap two arbitrarily selected periods (e.g., a cluster composed of 20 hourly GPS points might appear as of 10 if unintentionally bisected). Duplicate clusters were visually detected and removed from further analyses.

### 2.5 Field data collection

Field site visits at GPS locations were conducted in both 2006–2007 and 2013–2014, but with different field sampling designs. Both studies were on Grizzly Bears foraging and relied on GPS collars. However, the technological differences in the instrumentation available, which are explained above (data downloaded by aerial or ground‐based methods in 2006–2007 and via satellite uplink every 10 hr in 2013–2014), determined differences in the promptness of field visits by the project personnel.

During 2006–2007, we visited 464 daily randomly selected grizzly bear GPS locations following procedures from Munro et al. (2006). One location per day for each bear was examined (stratified random design) about 26 days (SD = 5) after the bear had visited that location.

Although clusters were detected the same way for both datasets, for the 2006–2007 period, clusters were generated *a posteriori* to assess whether a location fell within the start and end time of a generated cluster and within that same cluster's 50 m spatial constraint. If a cluster fit those criteria, the location was considered *within* a cluster (*n *=* *187), otherwise *outside* a cluster (*n *=* *277) (Figure 3).

For the 2013–2014 period, crews visited only sites falling within detected clusters (*n *=* *335, Figure 4). Following previous kill site studies (Cristescu et al., 2014a; Knopff et al., 2009; Pitman, Swanepoel, & Ramsay, 2012), we visited the largest two clusters per collared grizzly bear per week and then visited a random selection of the remaining generated clusters. For safety reasons, clusters were visited at least 5 days after the collared bear left the area and on average 11 days (SD = 10) after the start of the cluster.

When in the field, crews followed different protocols for data collection to assess the presence of predation events in 2006–2007 compared to 2013–2014. Our newer method benefited from combining rapid data download via satellites (not available for the older method) with the clustering analysis conducted in this study. Thus, we proceeded based on previous knowledge of clusters for 2013–2014 only. During 2006–2007, crews performed a meandering search near the selected GPS location. If grizzly bear sign was detected, it became the center of a defined 30 × 30 m plot search area.

In 2013–2014, clusters were explored by searching a 50‐m‐radius area starting from the GPS point closest to the geometric mean of the detected space–time cluster and spiraling outward. In previous studies, geometric centroids have been used as starting points (Knopff et al., 2009; Webb et al., 2008; Wilmers et al., 2013). However, this can lead to searching a location not actually occupied by the collared animal for clusters with large spatial spreads.

It can be difficult to rely on a carcass to determine cause or agent of death (e.g., predation, disease, winter kill). This is particularly true when carcasses are small due to complete consumption or scavenging (Franke et al., 2006; Webb et al., 2008). Bears are also capable of moving a carcass or stealing from another predator, which makes the identification of the original predator very complex. For these reasons, we marked all identified carcass locations as *predation events* when no substantial evidence of another predator was noted (e.g., scat, tracks), using established criteria by Hatter (1988) and Mattson (1997). Predation events were further classified according to binary prey size (large or small/medium). Large carcasses referred to only adult moose, and small/medium referred to all other identified remains (moose yearling, moose calf, deer, elk). Moose yearlings were distinguished from adults by tooth eruption and wear using guides from Northern Prairie Wildlife Research Center (U.S. Geological Survey 2006).

A location was classified as a predation event if we found prey remains that appeared to correspond to the time of the bear GPS location (i.e., sites with old, dried‐out bones were not included) and if there was clear sign of grizzly bear presence such as bear scat, tracks, hair, bedding (shallow to moderate depression or exposed soil, often hair is identified for verification) (Akenson, Nowak, Henjum, & Witmer, 2003; Munro et al., 2006; Podgórski, Schmidt, Kowalczyk, & Gulczyńska, 2008; Svoboda, Belant, Beyer, Duquette, & Martin, 2013), anting (logs over turned or opened, or ant hills unearthed) (Munro et al., 2006), and root digging (shallow to deep exposed holes) (Munro et al., 2006). Berry and herbaceous feeding sites were often difficult to identify depending on plant phenology and the plant species involved. For example, browsed buffaloberry can be quite obvious, whereas grazed clover can be inconspicuous. If a site contained substantial evidence of scavenging (wolf, cougar, or black bear behavior, scat or tracks) (Mattson, 1997) and minimal grizzly bear evidence, it was noted and classified as a *no predation event*, due to the likelihood that little or no feeding was performed at this site by a grizzly bear, despite the bear's presence. A typical site that we considered to be a grizzly predation was often where crews found a buried carcass, peeled hide, or, if located, a crushed skull.

### 2.6 Characterization of spatiotemporal clusters

In addition to the biological data, clusters were characterized by spatiotemporal characteristics: 1) *spread*: standard distance (i.e., standard deviation of the distance) (Mitchell, 2005) of GPS points of each cluster from its geometric center; 2) *number of GPS observations* (nGPSObs): GPS points per cluster; 3) *duration*: total time (hours) from first GPS observation to the last (the difference between *nGPSObs* and *duration* is due to the clustering algorithm not requiring GPS points to be consecutive to be considered within the same cluster. Therefore, if a grizzly bear returned to an already initiated cluster, the cluster would increase in size); 4) *occupation*: a ratio representing presence of an animal within an active cluster (GPS observations ÷ duration); 5) *starting time of day* (SToD): categorical variable indicating the time of day of the first GPS point in the defined cluster (categories were used from previous study on grizzly bear movement patterns (Munro et al., 2006) and daytime was used as reference for models); 6) *return events*: number of times the bear left and returned to the cluster within the duration of the cluster; 7) *season*: categorical variable indicating season divided as: 1 May to 15 June (Spring/hypophagia), 16 June to 15 August (Summer/early hyperphagia), and 16 August to 15 October (Fall/late hyperphagia; Fall was used as reference for models). These periods were chosen based on a previous grizzly bear study (Nielsen, 2005).

### 2.7 Univariate analysis

Pearson's chi‐square was used to compare the frequency of successfully located predation events in 2006–2007 between randomly visited grizzly bear GPS locations and those identified as STPSS clusters. For 2013–2014, we compared cluster sites with and without predation events, as well as sites between predation events containing small/medium vs. large prey carcasses, using Pearson's chi‐square for nominal variables (sex, age, season, time of the day of the start of the cluster) and Fisher's *t* test for continuous variables as number of GPS points, spread, duration, occupation, return events (Sokal & Rohlf, 1995).

### 2.8 Model formulation

Carcass size information was not available for the 2006–2007 dataset, because sites, when visited, often did not contain enough remains to indicate size of prey. Using just the 2013–2014 dataset of focused cluster visits (for which carcass size data were also available), we therefore formulated generalized linear mixed models (GLMM) (Dobson, 2008) to predict predation events or prey size using cluster spatiotemporal characteristics and bear sex and age as predictors. We used bear ID as a random effect in both models to account for multiple clusters from the same individuals with potentially unique behaviors (Gillies et al., 2006). Outcome (dependent) variables included: the presence or absence of a predation event, and the size of the carcass or prey (small/medium or large). We expected small/medium prey to be the usual outcome as predation on small prey is more difficult to detect.

Cluster characteristics (spatial spread, number of observations, duration, return events, and occupation), grizzly bear sex (male/female) and age (adult/subadult), plus interaction terms between bear age class × season and bear age class × sex were used as predictors (independent variables) in formulating our predictive models. The number of observations per cluster (log‐transformed) and duration (log‐transformed) were used to scale the variable for rare instances of prolonged site visits. We used these interaction predictors because previous studies have shown biased predation on ungulates by male and female grizzly bears (Boertje, Gasaway, Grangaard, & Kelleyhouse, 1988; Mattson, 1997; Reynolds & Garner, 1987). There have also been reports of different use of ungulate carcasses by adults compared to subadults (Young & Mccabe, 1997) as well as seasonal patterns of prey consumption by males and females (Milakovic & Parker, 2013).

We used an “all‐combinations” approach to select the three best‐performing models as ranked by the Akaike's information criterion (AIC) with a correction for small sample size (AICc) using the R statistical package MuMIn (Burnham & Anderson, 2002; R Core Team 2014). Among the best three models, we selected those with the highest sensitivity (proportion of correctly classified positive events: predation events or small/medium carcass). Model performance was also assessed by plotting the receiver operating characteristic curves (ROC) (Fielding & Bell, 1997) and by comparing sensitivity at multiple probability cutoff thresholds (0.5–0.9). ROCs were then tested using a nonparametric approximation (*W*; similar to a Mann–Whitney statistics) of the area under the curve and estimating its 95% confidence interval to verify that the observed curves were different than the ones that would have been obtained with a null model (*W *=* *0.5) (Liu et al., 2005).

## 3 RESULTS

### 3.1 Detecting predation with space–time clustering protocol

In 2006–2007, the probability of locating predation events was 0.058 (stratified random design, 27 predation sites, 464 visits, SE = 0.01). Of these 27 sites, 24 fell within *a posteriori*‐generated clusters (χ^2^ = 22.064, d.f.  = 1, *p *<* *.001) and predicted cluster centers had an average distance of 16.3 m (SE = 1.0) from carcasses. In 2013–2014, by selecting sites to visit among those falling into preliminary detected ST clusters, we increased by 2.75 times our ability to find predation/scavenging sites compared to the approach used in 2006–2007 (χ^2^ = 22.66, d.f.  = 1, *p *<* *.001). Overall, we identified predation sites with a 0.161 probability (54 predation events over 335 visits). Predicted cluster centers were an average of 16.0 m from carcass remains (SE = 2.2).

### 3.2 Characteristics of spatiotemporal clusters

Prey remains found during 2013–2014 consisted of 17 large prey (adult moose) and 37 small‐to‐medium‐sized prey (elk, deer, moose calf, moose yearling). While prey remains were only found at 16% of clusters, every cluster we visited contained evidence of at least one other type of grizzly bear activity. Of the visited clusters, 69% had sign of root digging, 68% bedding, and 22% anting. Sites characterized by herbivory and feeding were identified at 10 and 4% of clusters, respectively.

Cluster characteristics at predation sites showed some differences when compared to sites without predation (Tables 1 and 2). From our cluster visits, we found predation events were twice as likely during summer and fall than in spring (Table 1; *X* ^*2*^= 11.18, d.f.  = 2, *P*_Exact_ = 0.003). Clusters were five times more likely to begin during the day (32.8% of the cases) than at night (6.7%), and almost two times as likely than at twilight (18.2%; *X* ^*2*^ = 37.35, d.f.  = 2, *P*_Exact_ < 0.001; Table 1). No significant difference was detected between clusters of males and females relative to predation probability (18.4% vs. 12.5%; *X* ^*2*^ = 2.01, d.f.  = 1, *P*_Exact_ = 0.102).

**Table 1:** Spatiotemporal characteristics of predation events and carcass size around location clusters from GPS‐collared grizzly bears in west‐central Alberta

	Predation	No predation			Small/ Med carcass	Large carcass		
Variable	*n* (%)	*n* (%)	*X* ^2^	*P*_Exact_	*n* (%)	*n* (%)	*X* ^2^	*P*_Exact_
Season^a^								
Spring	17 (24.3)	53 (75.7)			11 (15.7)	6 (8.6)		
Summer	19 (22.6)	65 (77.4)	11.18	0.003	17 (20.2)	2 (2.4)	18.55	0.001
Fall	18 (9.9)	163 (90.1)			9 (5.0)	9 (5.0)		
SToD^b^								
Day	38 (32.8)	78 (67.2)			30 (25.9)	8 (6.9)		
Night	14 (6.7)	194 (93.3)	37.35	<0.001	6 (2.9)	8 (3.8)	43.38	<0.001
Twilight	2 (18.2)	9 (81.8)			1 (9.1)	1 (9.1)		
Sex^c^								
Male	38 (18.4)	169 (81.6)	2.01	0.102	24 (11.6)	14 (6.8)	3.52	0.170
Female	16 (12.5)	112 (87.5)			13 (10.2)	3 (2.3)		
Age class^d^								
Adult	43 (16.7)	214 (83.3)	0.31	0.360	27 (10.5)	16 (6.2)	3.22	0.221
Subadult	11 (14.1)	67 (85.9)			10 (12.8)	1 (1.3)		

Numbers and proportions (expressed as %) of sites found with and without evidence of a predation event by prey size, season, time of day, grizzly bear sex and age during searches conducted at GPS clusters in the Kakwa region (west‐central Alberta, Canada) in 2013–2014.

a Spring (1 May to 15 June), summer (16 June to 15 August), fall (16 August to 15 October) (Nielsen, 2005).

b Starting time of day, categorical variable of time of day of chronologically first GPS point in defined cluster (Munro et al., 2006).

c Sex of the collared grizzly bear.

d Age class of collared grizzly bear, Adult (≥4 years), subadult (<4 years) determined from premolar extraction.

**Table 2:** Continuous spatiotemporal characteristics of predation events and carcass size around clusters of locations from GPS‐collared grizzly bears in west‐central Alberta

Variable	Predation vs. No predation				Large vs. Small/Medium carcass			
	Mean (SE)		Students *t* test		Mean (SE)		Students t test	
	Predation (*n *= 54)	No predation (*n *= 281)	*t*	*p*‐value	Small/Med (*n *= 37)	Large (*n *= 17)	*t*	*p*‐value
Spread^a^	19.9 (1.0)	16.6 (0.4)	−3.11	.002	19.6 (1.1)	20.6 (1.9)	0.47	.641
nGPSObs^b^	24.7 (3.0)	7.1 (0.2)	−5.85	<.001	20.5 (2.3)	32.9 (7.6)	1.58	.131
Duration^c^	41.1 (6.1)	18.8 (2.7)	−3.36	.001	37.8 (6.6)	47.7 (12.9)	0.68	.502
Return events^d^	1.8 (0.2)	0.4 (0.1)	−5.18	<.001	1.75 (0.3)	2 (0.6)	0.38	.706
Occupied^e^	0.7 (0.0)	0.8 (0.0)	2.28	.026	0.72 (0.0)	0.79 (0.1)	0.80	.429

Means and standard errors of the GPS point number, spread, duration, return events, and proportion of occupation within clusters found with and without evidence of a predation event by prey size during searches conducted in the Kakwa region (west‐central Alberta, Canada) in 2013–2014.

a “Standard distance” of GPS points in cluster (m).

b Hourly GPS points per cluster.

c Total time (h) from first cluster GPS observation to last observation.

d Number of times the grizzly bear left and returned to the cluster within the duration of the cluster.

e Proportion of animal presence within active cluster (GPS observations ÷ cluster duration).

Clusters around a predation event had larger spread (19.9 vs. 16.6 m, *t *= −3.11, *p *=* *.002), contained more GPS locations (24.7 vs. 7.1 points, *t *= −5.85, *p *<* *.001), and lasted twice as long (41.1 vs. 18.8 hr, *t *= −3.36, *p *<* *.001) than clusters where no predation event was detected. Bears were also more likely to leave and then return to a predation than a nonpredation event cluster with an average of 1.8 return events per predation event cluster (*t *= −5.18, *p *<* *.001 l; Table 2).

We detected a seasonal pattern when we compared predation events that had small/medium prey carcasses to those where larger prey carcasses were found (Tables 1 and 2). In spring, clusters were more likely to indicate large than small/medium carcasses (ca. 65% vs. 35%, *X* ^2^ = 18.55, *p *=* *.001) than in summer and fall when most of the clusters we detected were around small/medium prey (89.4% and 64.7%, respectively).

### 3.3 Predictive model

#### 3.3.1 Predation event model

**Figure 5:**
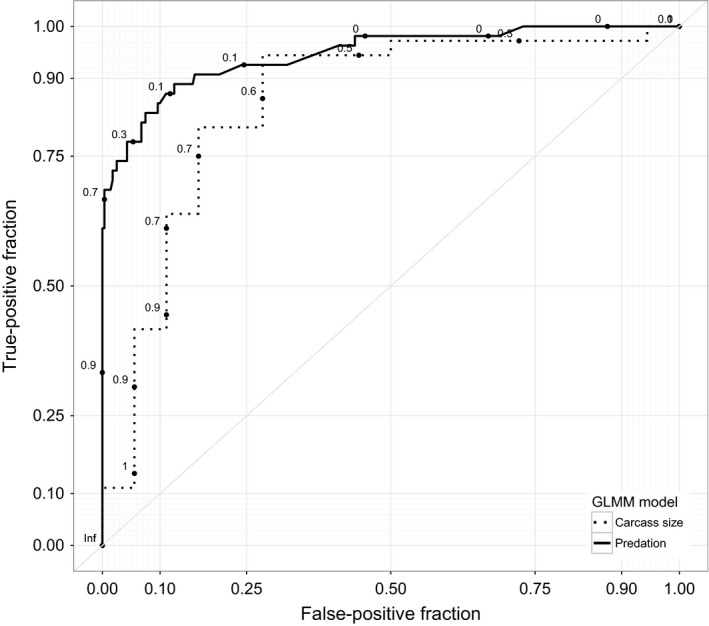
Receiver operating characteristic (ROC) curves of the generalized linear mixed models (GLMM) used to predict grizzly bear predation from clusters (solid line) and prey size when predation occurs (dotted line). These curves show true‐positive versus false‐positive successful predictions and apply to clusters visited during 2013–2014. The area under the curve (AUC) for these lines are 0.945 (solid line) and 0.852 (dotted line)

The best model included four variables: total number of GPS points in the cluster, cluster duration, sex of the grizzly bear, and cluster starting time (Table 3). The likelihood of finding a predation event increased with the number of GPS observations in the cluster, if the bear was male and when the cluster started during the day. A cluster starting during night or at twilight gave a negative effect (Table 4). Using the usual probability threshold of 0.5, the model had an excellent overall classification success (93.4% combined correct classification of “predation” and “no predation”), good sensitivity (72.2% correctly classified cases of “predation”), and very high specificity (97.5% correctly classified cases of “no predation”) with an expected decrement as cutoff thresholds increased (Table S3). The estimated area under the curve (AUC) for the top model was 0.945 (SE = 0.018, *p *<* *.001) and the ROC plot indicated minimal false positivity in prediction (Figure 5).

**Table 3:** Top‐ranked binomial logistic regression models for predicting predation events and carcass size developed on clusters of locations from GPS‐collared grizzly bears in west‐central Alberta

Rank	Variables	LL	K	AIC_c_	Δ_*i*_	ω_*i*_	Sensitivity (in %)
Predation models							
1	nGPSObs^a^ + duration^b^ + SToD^c^ + sex^d^	−60.5	7	135.3	0.0	0.076	72.2
2	nGPSObs + SToD + sex	−61.9	6	136	0.71	0.053	72.2
3	nGPSObs + occupied^e^ + sex + SToD	−61.1	7	136.5	1.18	0.042	72.2
Carcass size models							
1	season^f^ + SToD + nGPSObs + spread^g^	−25.0	8	69.3	0.86	0.018	94.4
2	season	−29.8	4	68.4	0.0	0.027	77.8
3	season + SToD	−27.6	6	69.0	0.61	0.020	77.8

Top‐ranked binomial logistic regression models for predicting predation sites by prey size developed from visited clusters of GPS locations from collared grizzly bears in west‐central Alberta, Canada in 2013–2014. Models contain grizzly bear ID as a random effect and are shown in decreasing rank by Akaike's Information Criterion with a correction for small sample size (AIC_c_) and compare log‐likelihood (LL), number of estimated parameters (K), AIC*c* difference (Δ_*i*_), AIC_c_ weight (ω_*i*_) and the model's classification specificity as a percentage of correctly classified positive results.

a Hourly GPS points per cluster.

b Total time (h) from first cluster GPS observation to last observation.

c Starting time of day, categorical variable of time of day of chronologically first GPS point in defined cluster (Munro et al., 2006).

d Sex of the collared grizzly bear.

e Proportion of presence of animal within active cluster (GPS observations ÷ cluster duration).

f Spring (1 May to 15 June), summer (16 June to 15 August), fall (16 August to 15 October) (Nielsen, 2005).

g “standard distance” of GPS points in cluster in meters (ESRI, Redlands, Calif).

**Table 4:** AICc selected best predictive binomial logistic regression models for predation for determining the size of a carcass at clusters of locations from GPS‐collared grizzly bears in west‐central Alberta

	Estimate	SE	*z*	*p*‐value	Odds ratio	Lower 95% Conf. Int.	Upper 95% Conf. Int.
a) Predation detection							
Intercept	−10.75	1.49	−7.07	<.0001	2.5456^g^ 10^−5^	8.3540 g 10^−7^	0.0004
nGPSObs^a^	11.24	1.83	6.14	<.0001	7.5904^g^ 10^4^	3.0228 g 10^3^	4.4186^g^ 10^6^
Duration^b^	−1.46	1.83	6.14	.1229	0.0233	0.0292	1.2549
Sex(M)^c^	1.32	0.54	2.44	.0149	3.7373	1.3117	13.8550
SToD^d^	–	–	–	<.0001^g^	–	–	–
Night	−2.45	0.52	−4.74	<.0001	0.0859	0.0294	0.2275
Twilight	−3.21	1.70	−1.89	.0594	0.0404	0.0013	0.6280
b) Carcass size determination							
Intercept	4.80	2.25	2.13	.0331	120.9318	1.8911	1.6273^g^ 10^4^
Season^e^	–	–	–	.0134^g^	–	–	–
Spring	0.54	0.81	0.66	.5083	1.7101	0.3487	8.8467
Summer	2.65	1.06	2.51	.0123	14.1922	2.1467	156.1204
SToD	–	–	–	.0424^g^	–	–	–
Night	−1.91	0.86	−2.23	.0261	0.1476	0.0234	0.7384
Twilight	−1.92	1.69	−1.14	.2564	0.1466	0.0037	5.3472
nGPSObs^e^	−1.97	1.20	−1.64	.1018	0.1396	0.0108	1.3911
Spread^f^	−3.48	2.07	−1.68	.0926	0.0308	0.0003	1.3687

Selected models for predicting a) cluster sites containing predation, and b) the size of a carcass given that predation is present using clusters of GPS locations from collared grizzly bears in west‐central Alberta, Canada in 2013–2014. Coefficient estimates are shown with standard errors, Wald statistics (*z*) associated *p* values, the odds ratios and upper and lower confidence intervals (CI) of the odds ratios. Categorical variable SToD had daytime withheld and season had fall withheld as reference variables.

a Hourly GPS points per cluster.

b Total time span (hours) from first cluster GPS observation to last observation.

c Sex of the collared grizzly bear, male was the reference variable, that is, value = 1.

d Starting Time of Day(SToD), categorical variable of time of day of chronologically first GPS point in defined cluster, daytime was withheld as a reference variable (Munro et al., 2006).

e Season: spring (1 May to 15 June), summer (16 June to 15 August), fall (16 August to 15 October), fall was withheld as a reference variable (Munro et al., 2006).

f “Standard distance” of GPS points in cluster in meters (ESRI, Redlands, Calif).

g Overall effect significance estimated using ANOVA with/without categorical variable in question.

#### 3.3.2 Carcass size model

We used the carcass size model with high sensitivity (94.4%) despite a slightly lower AIC_c_ (Table 3). For the carcass size predictive model, season (summer) and SToD (night) were found to be significant predictors of small/medium prey carcasses (*p *≤* *.05) whereas the number of GPS points and spread were not significant individually but increased the AIC_c_ of the model and including these variables improved overall model success (Table 3).

Similar to the predation model, using a probability cutoff of 0.5, the best‐performing GLMM model for predicting carcass size had a very high classification rate (85.2%; Table S3) with a very high sensitivity (94.4% of correctly classified positive cases) and a good specificity (66.7% of correctly classified negative cases) with an expected decrement as cutoff thresholds increased (Table S3).

As with the previous model, the estimated area under the curve (AUC) for this model was very high at 0.852 (SE = 0.062, *p *<* *.001) and the ROC plot indicated minimal false positivity in prediction (Figure 5) (Hosmer, Lemeshow, & Sturdivant, 2013).

The likelihood of finding a small/medium carcass at a predation site was greater in fall and summer (Table 4) and increased when the cluster started during the night or at twilight. The number of GPS locations in the cluster, and the size of the cluster, although important to increase the predictive power of the model, did not individually affect the probability of finding a small/medium carcass.

## 4 DISCUSSION

Our method, based on an advanced spatiotemporal algorithm (space–time permutation scan statistic) used on almost real‐time GPS data to detect predation event clusters, allowed for early identification (within two weeks from cluster) and proved to be very efficient in detecting grizzly bear predation events in west‐central Alberta. While there are computational and logistical limitations to this approach, we argue that this result is a significant advance in our ability to measure predation rates, prey characteristics, and predator behavior.

When applied to randomly selected daily GPS locations, our clustering methods successfully identified grizzly bear predation events 85.1% of the time. Only four carcasses in the 2006–2007 dataset were not identified as predation events. The first two were moose calves, the third was likely a scavenging event, and the fourth was an adult moose. While not all of these misclassifications can be directly accounted for, even with short‐interval GPS data (Cristescu et al., 2014b; Webb et al., 2008) small prey and scavenging events can go undetected due to the short time it takes to consume or move the carcass (Cavalcanti & Gese, 2010; Webb et al., 2008).

Using our cluster‐oriented approach, we increased the probability of finding a carcass site by 2.75 times. The probability increased from 0.058 carcasses per site visit in 2006–2007 to 0.161 in 2013–2014. While this might be attributed to simply a difference in predation patterns between sampling periods, we suggest that a result of this magnitude represents an improvement in detection between the two datasets due to the clustering method combined with quick site visits.

Early detection of predation events (mean = 11 days) represented a substantial improvement over previous studies. Cristescu et al. (2014b) was limited by how frequently they downloaded GPS data using remotely downloadable GPS radiocollars. Monthly downloading resulted in visiting sites on average 3 weeks after the kill event. Bacon, Becic, Epp, and Boyce (2011) downloaded data *via* ground telemetry every 3 weeks making sites at least that old when visited. Tambling et al. (2012) examined African lion predation with a median of 24 days after the animal left the site. Webb et al. (2008) searched wolf GPS clusters with a delay of up to 45 days after the wolves had been at the cluster and discussed the potential impact of such delays on studies where predators relied on small‐bodied prey. One of the original GPS clustering studies (Anderson & Lindzey, 2003) visited sites an average of 201 days after the event. Their study was limited by the use of store‐on‐board collars, was unable to distinguish predation from scavenging, and had limited ability to detect smaller prey or to determine sex and age of the carcass. Our success in reducing delays increases the information gained from predation events, increasing the efficiency of field efforts and boosting predation event sample size. The value of this is greater when the focal predator is an omnivore because predation and subsequent feeding is often a small fraction of their overall activity budget.

By exploring the spatiotemporal characteristics of grizzly bear behavior we found, as have others for bears and other carnivores (Anderson & Lindzey, 2003; Cristescu et al., 2014b; Ebinger et al., 2016; Franke et al., 2006; Knopff et al., 2009; Rauset et al., 2012; Sand et al., 2005; Tambling et al., 2010; Webb et al., 2008), that predators spend more time around predation sites. Cluster characteristics around predation events were distinct from those without predation allowing for a successful detection of the predation event. Both the number of GPS points and the duration of a cluster provide insight into how a bear spends time at a predation site. The more time the grizzly bear was at the location, the greater probability of it being predation. This is consistent with findings from other studies regardless of the predator species (Anderson & Lindzey, 2003; Cavalcanti & Gese, 2010; Cristescu et al., 2014b; Knopff et al., 2009; Tambling et al., 2010; Webb et al., 2008).

The time of day the cluster was initiated (SToD) strongly increased the predictive power of our models. While this does not necessarily indicate the beginning of a predation event, clusters initiated during the day were significantly more likely to indicate the presence of a carcass. This may indicate that killing or scavenging is less likely at night or twilight when other behaviors, such as bedding, may be dominant (Munro et al., 2006). This relationship agrees with similar research in our study area (Cristescu et al., 2014b; Stenhouse & Munro, 2000) as well as in North America (Craighead, Sumner, & Mitchell, 1995) that has shown that grizzly bears are active primarily during the day (Graham & Stenhouse, 2014). However, these results differ from obligate carnivores such as wolves that tend to hunt more actively during crepuscular hours (Theuerkauf et al., 2003) and cougars which are more active at night than during the day (Kertson, Spencer, Marzluff, Hepinstall‐Cymerman, & Grue, 2011).

Interestingly, predator gender significantly affected the likelihood of detecting predation events. We found higher detection likelihood for male grizzly bears. This finding is consistent with research indicating meat consumption and predation among grizzly bears is higher in males than females (Boertje et al., 1988; Jacoby et al., 1999; McLellan, 2011). However, this is in contrast with López‐Alfaro, Robbins, Zedrosser, and Nielsen (2013) who suggested that females would have large benefits from increased meat consumption.

Models using GPS data have not been very successful in predicting or detecting small‐bodied carcass sites (Ebinger et al., 2016; Webb et al., 2008). This is likely due to the short handling time required to eat small prey and thus a less conspicuous spatiotemporal cluster. However, with our approach, we successfully detected small carcasses, identifying 37 small/medium‐bodied prey (Table S2), with high model sensitivity.

By improving small‐prey detection, we observed marked seasonality in their use by grizzly bears with higher frequency in fall and summer. This is contrary to previous studies that found smaller prey in spring. These studies suggested that this pattern was due to both the increased vulnerability of young ungulates (Munro et al., 2006; Rauset et al., 2012), and grizzly bears’ propensity to feed on carcasses in spring due to increased availability of alternative high‐energy plant foods (Boertje et al., 1988). Although there can be increased availability of plant food in summer, there is also a dietary need for high‐density protein found in meat. Many studies have shown that despite this increased access to plant protein, a high‐density grizzly population depends on access to high‐protein meats (Miller et al., 1997; Rode, Robbins, & Shipley, 2001; Welch, Keay, Kendall, & Robbins, 1997). Moreover, our model showed an increased likelihood of small/medium carcass detection in the fall is consistent with an overall increased predation rate. This is possibly linked to an increased need for protein during the predenning period and potentially due to an increase in prey mobility as they age (Ballard, Gardner, & Miller, 1980; McLellan & Hovey, 1995; Munro et al., 2006).

The success of modeling predation events using predator GPS collar data has varied by predator and prey species being studied (Anderson & Lindzey, 2003; Franke et al., 2006; Knopff et al., 2009; Sand et al., 2005; Tambling et al., 2010; Webb et al., 2008). This approach is relatively new to omnivores such as grizzly bears (Cristescu et al., 2014b; Rauset et al., 2012). While Rauset et al. (2012) experienced comparable success using an alternate method that relies on logistic regression, their study applied strictly to female grizzlies during the early spring calving season. Our predation event detection was slightly increased compared to Cristescu et al. (2014b) who found around 80% identification success of ungulate carcasses. Varying success from each of these studies may also result from differences in GPS fix rates and differences in feeding patterns among individual grizzly bears. We were able to download data every ten hours giving us greater resolution in grizzly bear feeding behavior. Successful prediction is more likely associated to shorter delays in visiting clusters.

Predicting prey size at detected predation events is a new and developing research field. Cristescu et al. (2014a), while successful at predicting the location of prey cached by grizzlies, failed to predict the size of prey. Webb et al. (2008) were also challenged when attempting to predict prey size for wolf kill sites. Ebinger et al., 2016 applied an *a posteriori* spatial cluster detection method to describe clustering behavior only around selected sites using a daily stratified random sampling approach (as the one we used for 2006–2007 seasons). They visited sites less than 10 days after GPS data collection and successfully classified clusters around large carcasses, but had poor sensitivity (43–55%) in identifying small carcass sites.

Efficient field sampling is critical to ensure that sufficient and accurate data are collected with limited resources. Our study provides an effective alternative to the most commonly used approaches to explore predation rate in obligate or nonobligate carnivores. While a project might benefit from visiting all clusters or a large number of randomly selected GPS points to provide a better estimate of grizzly bear predation events, the substantial field investment and related costs make such approaches less desirable. This is particularly true for omnivorous or nonobligate carnivore species. For obligate carnivores, clustering behavior more reliably indicates a predation event (Anderson & Lindzey, 2003; Webb et al., 2008), whereas omnivorous/nonobligate carnivore clustering is less clearly indicative of a predation event. Most often, their time is allocated to other activities such as grazing (Cristescu et al., 2014b) or anting. The large number of sites that must be visited to achieve this level of resolution in the absence of effective prediction remains a logistical and resourcing challenge.

We attribute our success in predicting predation events and the size of the prey to several factors. The short delay between collar data collection and site visits is clearly important. But we believe a vital element to success is selecting clusters using a preliminary identification algorithm. This greatly improved the likelihood of including sites where predation actually occurred. Moreover, the use of an advanced algorithm based on a permutation approach (STPSS) to compare observed versus expected spatiotemporal distribution of GPS location significantly improved our ability to detect important behaviors on the field.

It is important to stress that these algorithms rely on an appropriate GPS fix rate and require the constraints imposed by the clustering algorithm to be consistent with the behavior of the studied predator around a food source. In our case, we could rely on numerous studies that provided estimates of these parameters (Anderson & Lindzey, 2003; Cristescu et al., 2014b; Franke et al., 2006; Knopff et al., 2009; Rauset et al., 2012; Sand et al., 2005; Tambling et al., 2010; Webb et al., 2008). For these reasons, when planning a predation behavior study in carnivores, obligate or not, we recommend paying careful attention to the GPS fix rate as it relates to success in identifying predation events. Future studies should additionally resist sampling specific seasons due to the link we found between the size of prey carcasses and season. Ultimately, we would recommend the use of STPSS clusters for identifying kill sites. But equal priority should be placed on species‐specific analyses to accurately estimate algorithm parameters.

## CONFLICT OF INTEREST

None declared.

## AUTHORS CONTRIBUTIONS

JKM collected field data on clusters and performed data analyses; AM coordinated and supervised data analyses and hypothesis formulation and MS writing; SG and LT coordinated, implemented, and collected radiotelemetry of grizzly bears and discussed methodologies and data analyses; MM coordinated the overall project and supervised the data analyses and hypothesis formulation.
