# Supplementary material for: Space–time clusters for early detection of grizzly bear predation
Source: Ecol Evol. 2017 Nov 29;8(1):382–95. doi: 10.1002/ece3.3489 (PMC5756826; doi:10.1002/ece3.3489)
Supplement: Supplementary file 1 [file ECE3-8-382-s001.docx]

**Supporting Information**

26 June, 2017

J. Kermish-Wells, A. Massolo, G.B. Stenhouse, T. Larsen, & M. Musiani. 2017. Space-time clusters for early detection of grizzly bear predation. Methods in Ecology and Evolution: in review.

Table S1. Age classes and sexes of grizzly bears tagged with GPS collars for 2006-2007 and 2013-2014 seasons (May to October) in the Kakwa region in west-central Alberta (Canada). Adult (≥ 4 years), subadult (< 4 years) determined from molar extraction.

|  | Adult | SubAdult | Total |
| --- | --- | --- | --- |
| 2006-2007 |  |  |  |
| Male | 0 | 2 | 2 |
| Female | 6 | 1 | 7 |
| Total | 6 | 3 | 9* |
| 2013-2014 |  |  |  |
| Male | 5 | 1 | 6 |
| Female | 3 | 1 | 4 |
| Total | 8 | 2 | 10* |

*One bear was collared from 2006-2013, and used separately in each analysis. Therefore, n = 18 bear GPS collar data were used.

Table S2. Contains the modifications to the default parameters used by (Kulldorff *et al.* 2005) as recommended by previous SaTScan ecological studies (Webb *et al.* 2008; Pruvot *et al.* 2014). The analysis used for this study was retrospective space-time permutation.

| Parameters | Default | Selection/modification |
| --- | --- | --- |
| Time Precision | None; Year; Month; Day; Generic | Generic (hours) |
| Study Period | Start time: End time | 2 week period of generic units (hours) |
| Case file | Locations can have multiple cases | Locations are independent, 1 case for each location |
| Scan For Areas With: | High Rates; Low Rates; High or Low Rates | High Rates |
| Time aggregation | Length of the time intervals in specified units. | 1 hour (4 units) |
| Is a circle with radius | Unchecked | Checked: 0.05 kilometer radius |
| Maximum Temporal Radius | 50% of the study period | 50% of the study period |
| Montecarlo Replications | 9,99,999 or value ending in 999 | 999 |

Table S3. Confusion matrices for a range of thresholds used to determine classification success (0.5-0.9) of GLMM models used to predict predation events and size of carcasses. Sites were visited from May to October in 2013-2014 in the Kakwa region in west-central Alberta, Canada. ‘Predation’ and ‘no predation’ refer to the contents of the visited locations and ‘Small/Med’ and ‘Large” refer to the size class of identified carcass remains. Bolded values indicate correct predictions.

|  | | **Predation vs No Predation** | | |  | | **Small/Med vs Large Carcasses** | | |
| --- | --- | --- | --- | --- | --- | --- | --- | --- | --- |
| 0.50 |  | | PREDICTED [n (%)] | |  |  | | PREDICTED [n (%)] | |
|  |  | |  |  |  |  | |  |  |
|  |  | | No Predation | Predation |  |  | | Large | Small/Med |
| REAL | No Predation | | **274 (97.5)** | 7 (2.5) | REAL | Large | | **12 (66.7)** | 6 (33.3) |
|  | Predation | | 15 (27.8) | **39 (72.2)** |  | Small/Med | | 2 (4.6) | **34 (94.4)** |
|  | **Total 93.4%** | | | |  | **Total 85.2%** | | | |
| 0.60 |  | | PREDICTED [n (%)] | |  |  | | PREDICTED [n (%)] | |
|  |  | | No Predation | Predation |  |  | | Large | Small/Med |
| REAL | No Predation | | **276 (98.2)** | 5 (1.8) | REAL | Large | | **13 (72.2)** | 5 (27.8) |
|  | Predation | | 15 (27.8) | **39 (72.2)** |  | Small/Med | | 5 (13.9) | **31 (86.1)** |
|  | **Total 94.0%** | | | |  | **Total 81.2%** | | | |
| 0.70 |  | | PREDICTED [n (%)] | |  |  | | PREDICTED [n (%)] | |
|  |  | | No Predation | Predation |  |  | | Large | Small/Med |
| REAL | No Predation | | **280 (99.6)** | 1 (0.4) | REAL | Large | | **15 (83.3)** | 3 (16.7) |
|  | Predation | | 18 (33.3) | **36 (66.7)** |  | Small/Med | | 13 (36.1) | **23 (63.9)** |
|  | **Total 94.3%** | | | |  | **Total 70.4%** | | | |
| 0.80 |  | | PREDICTED [n (%)] | |  |  | | PREDICTED [n (%)] | |
|  |  | | No Predation | Predation |  |  | | Large | Small/Med |
| REAL | No Predation | | **281 (100)** | 0 (0) | REAL | Large | | **16 (88.9)** | 2 (11.1) |
|  | Predation | | 24 (44.4) | **30 (55.6)** |  | Small/Med | | 18 (50.0) | **18 (50.0)** |
|  | **Total 92.8%** | | | |  | **Total 63.0%** | | | |
| 0.90 |  | | PREDICTED [n (%)] | |  |  | | PREDICTED [n (%)] | |
|  |  | | No Predation | Predation |  |  | | Large | Small/Med |
| REAL | No Predation | | **281 (100)** | 0 (0) | REAL | Large | | **17 (94.4)** | 1 (5.6) |
|  | Predation | | 34 (63.0) | **20 (37.0)** |  | Small/Med | | 21 (58.3) | **15 (41.7)** |
|  | **Total 89.9%** | | | |  | **Total 59.3%** | | | |

Table S4. Distribution of carcasses fed on by bear. Separated into species/age classes of prey, and by season. Spring, summer, fall determined by spring (1 May to 15 June), summer (16 June to 15 August ), fall (16 August to 15 October) (Nielsen 2005).

|  |  | **Season** |  |  |
| --- | --- | --- | --- | --- |
| **BearID** | **fall** | **spring** | **summer** | **Grand Total** |
| **G260** |  | **1** |  | **1** |
| AdultFemaleMoose |  | 1 |  | 1 |
| **G270** | **2** | **3** | **5** | **10** |
| AdultFemaleMoose | 1 |  | 1 | 2 |
| AdultUnknownMoose |  | 1 |  | 1 |
| CalfMoose | 1 | 1 | 4 | 6 |
| YearlingMoose |  | 1 |  | 1 |
| **G275** | **1** | **2** | **3** | **6** |
| AdultUnknownMoose |  | 1 |  | 1 |
| CalfMoose |  | 1 | 3 | 4 |
| YearlingMoose | 1 |  |  | 1 |
| **G280** | **2** | **4** | **1** | **7** |
| AdultUknownUngulate | 1 |  |  | 1 |
| AdultUnknownMoose |  | 1 |  | 1 |
| CalfMoose | 1 | 2 | 1 | 4 |
| YearlingMoose |  | 1 |  | 1 |
| **G284** | **3** | **1** | **2** | **6** |
| AdultUnknownMoose | 1 |  |  | 1 |
| AdultWhitetaildeer | 2 |  |  | 2 |
| CalfMoose |  | 1 | 2 | 3 |
| **G286** |  | **1** | **1** | **2** |
| AdultFemaleMoose |  |  | 1 | 1 |
| CalfMoose |  | 1 |  | 1 |
| **G287** |  | **1** | **3** | **4** |
| CalfMoose |  | 1 | 1 | 2 |
| YearlingMoose |  |  | 2 | 2 |
| **G289** | **5** | **2** |  | **7** |
| AdultFemaleMoose | 1 |  |  | 1 |
| AdultMaleMoose | 1 |  |  | 1 |
| AdultUnknownMoose | 1 | 1 |  | 2 |
| CalfMoose | 1 | 1 |  | 2 |
| YearlingMoose | 1 |  |  | 1 |
| **G291** | **5** | **1** | **3** | **9** |
| AdultFemaleMoose | 2 | 1 |  | 3 |
| AdultMaleMoose | 2 |  |  | 2 |
| CalfMoose |  |  | 2 | 2 |
| ElkCalf | 1 |  |  | 1 |
| YearlingMoose |  |  | 1 | 1 |
| **G292** |  | **1** | **1** | **2** |
| CalfMoose |  | 1 | 1 | 2 |
| **Grand Total** | **18** | **17** | **19** | **54** |

**Supporting Information References**

Kulldorff, M., Heffernan, R., Hartman, J., Assunção, R. & Mostashari, F. (2005). A space-time permutation scan statistic for disease outbreak detection. *PLoS medicine*, **2**, 0216–0224.

Nielsen, S.E. (2005). *Habitat ecology, conservation, and projected population viability of grizzly bears (Ursus arctos L.) in west-central Alberta, Canada*. University of Alberta.

Pruvot, M., Seidel, D.P., Boyce, M.S., Musiani, M., Massolo, A., Kutz, S. & Orsel, K. (2014). What attracts elk onto cattle pasture? Implications for inter-species disease transmission. *Preventive veterinary medicine*, **117**, 326–339.

Webb, N.F., Hebblewhite, M. & Merrill, E.H. (2008). Statistical methods for Identifying wolf kill sites using global positioning system locations. *The Journal of Wildlife Management*, **72**, 798–807.
